# Interaction between GDF5 gene polymorphisms and environment factors increased the risk of knee osteoarthritis: a case–control study

**DOI:** 10.1042/BSR20182423

**Published:** 2019-02-26

**Authors:** Sujie Zhang, Juan Wang, Hongliang Ji, Helei Jia, Dongsheng Guan

**Affiliations:** 1 Department of Orthopedics, Dongzhimen Hospital Beijing University of Chinese Medicine, Beijing 101100, China; 2 The First Clinical College, Henan University of Chinese Medicine, Zhengzhou 450046, Henan Province, China; 3 Department of Endocrine and Cardiovascular, People’s Hospital of Huiji District, Zhengzhou 450044, Henan Province, China; 4 Department of Emergency, Henan Province Hospital of Traditional Chinese Medicine, Zhengzhou 450002, Henan Province, China

**Keywords:** GDF5, Hardy-Weinberg, gene polymorphism, knee osteoarthritis

## Abstract

Using a case–control design, we assessed the association between single nucleotide polymorphisms (SNPs) of growth and differentiation factor 5 (GDF5)/rs143383 gene and interaction with environments and knee osteoarthritis (KOA). We recruited 288 KOA patients from the First Clinical College, Henan University of Chinese Medicine between June 2017 and May 2018. There was significant difference in genotype distribution between case group and control group (χ^2^ = 22.661, *P*=0.000). The minor C allele was significantly higher in the case group than that in the control group (20.5 vs 8.1%, *P*=0.000, odds ratio (OR) = 1.62, 95% confidence interval (CI): 1.29–2.03). Significant differences were also observed in other gene models. For age, all models show significant differences (*P*<0.05) for those whose age was more than 60 years, and no significant difference was observed for those under 60 years. For non-smoking group, there were significant differences between case group and control group, and for smoker, significance level was found in TT compared with CC and allele gene models. Patients with drinking and Bbody mass index (MI )≥ 24 also showed significant relationship between rs143383 and osteoarthritis (OA) under the following models: TT vs CC (*P*=0.000, *P*=0.018), TT/CT vs CC (*P*=0.043), TT vs CT/CC (*P*=0.000, *P*=0.009), and T vs C (*P*=0.024, *P*=0.000). Other gene models indicated no significance (*P*>0.05). Our results revealed a possible genetic association between GDF5 and KOA, and the TT genotype of rs143383 increased the risk of KOA in Chinese Han population. The interaction between *GDF5* gene and drinking, smoking, and obesity further increased the risk of KOA.

## Introduction

Osteoarthritis (OA), the most prevalent chronic joint disease, increases in prevalence with age, and affects most individuals over the age of 65 and is a leading musculoskeletal cause of impaired mobility in the elderly [1,2]. According to a report, the expenses on OA treatments and other things related is approximately 1–2.5% of the GDP in developed countries [3]. OA mainly affects the joints including knees, hands, hips, and spine and is a leading musculoskeletal cause of impaired mobility in the elderly. The major clinical symptoms include chronic pain, joint instability, stiffness, joint deformities, and radiographic joint space narrowing [4]. Knee OA (KOA) is a serious rheumatic disease characterized by articular cartilage damage and joint space narrowing [5]. Treatments of OA involve alleviating pain, reducing stiffness, maintaining the functional capacities, and improving the quality of life. Current treatments include low-impact aerobic exercise, weight loss, acupuncture, glucosamine and chondroitin sulphate, and surgical [6]. Because the precise molecular mechanisms implicated in pathogenesis of KOA are poorly understood and there are currently no effective interventions to decelerate the progression of KOA or retard the irreversible degradation of cartilage except for total joint replacement surgery. Several risk factors associated with KOA have been put forward, including a genetic predisposition, ageing, obesity, dieting, and joint malalignment, the pathogenesis of KOA remains largely unclear [7–12].

The single nucleotide polymorphism (SNP) is more than 90% of human gene polymorphisms, which is the most common and stable gene variant in the human DNA strands [13]. The coding SNP consisted of synonymous coding SNP and non-synonymous coding SNP. Research has demonstrated that genetic factors contribute enormously in the etiology and pathogenesis of KOA. Growth and differentiation factor 5 (GDF5), which was also known as cartilage-derived morphogen protein of the transforming growth factor-β (TGF-β) superfamily, was shown to play a substantial role in the process of development, maintenance, and repair of cartilage and bone [14]. Polymorphisms in the *GDF5* gene have been involved in several skeletal developmental disorders, such as different forms of chondrodysplasia, symphalangism, and brachydactyly type C [15–17]. The variations in genetic susceptibility in different ethnic groups has led us to attempt to examine the association between GDF5 gene polymorphism (rs143383) and KOA. Currently, there is no report about the relationship between GDF5/rs143383 gene and KOA. The present study investigates the association between SNPs of GDF5/rs143383 gene and KOA in Chinese Han population.

## Materials and methods

### Study population

Using a case–control design, we recruited 288 KOA patients (129 males and 159 females) from our hospital between June 2017 and May 2018. We selected health controls without the history of OA and autoimmune diseases from physical examination center during the same period. The diagnostic criteria of KOA were performed by a rheumatologist according to the 2010 American College of Rheumatology/European League against Rheumatism criteria [18]. Diagnosis of KOA often entails a physical examination, assessment of symptoms, and the patient’s medical history, but may also involve medical imaging and blood tests. Persistent knee pain, limited morning stiffness and reduced function, crepitus, restricted movement, and bony enlargement appear to be the most useful indications of KOA for diagnosis. Patients with a history of knee or hip surgery, secondary OA, severe cardiovascular diseases, severe liver and kidney dysfunction, malignant tumor, and other autoimmune diseases were excluded from the study. Grades 3–4 of radiographic signs of OA were in accordance with the Kellgren—Lawrence grading system [19]. We using Quanto 1.2.4 to calculate the sample size and followed by the conditions: α = 0.05, β = 0.20, expected odds ratio (OR) = 1.8, the calculated sample size is 267 in the case group and control group, respectively. The present sample size is enough. The present study was approved by the Institutional Review Board of Henan University of Chinese Medicine. Written informed consent was received from all study subjects. The research has been carried out in accordance with the World Medical Association Declaration of Helsinki, and that all subjects provided written informed consent.

### Clinical data collection

We used a standard questionnaire to collect the clinical characteristics of study population. The collected information included age, gender, history of smoking and drinking, height and weight for body mass index (BMI) calculation (BMI is defined as the body mass divided by the square of the body height, and is universally expressed in units of kg/m^2^, resulting from mass in kilograms and height in meters), and Kellgren–Lawrence grading. The biological indicator included erythrocyte sedimentation rate (ESR) and C-reactive protein (CRP). Hematologic testing was conducted on the Beckman Coulter LH-750 Hematology Analyzer (Beckman Coulter, Inc., Fullerton, CA, U.S.A.), automated hematology analyzer, which measures ESR and CRP. According to the criteria: ESR ≥ 20 and CRP > 8 were judged as abnormal [20].

### DNA extraction

Approximately 2–3 ml of venous blood was collected by a sterile venipuncture using a sterile EDTA vacutainer. The blood samples were put in EDTA anticoagulant tubes, stored in the refrigerator at −20°C until use. Genomic DNA was extracted from EDTA anti-coagulated whole blood using QIAamp DNA Mini Kit (QIAGEN; Hilden, Germany) according to the manufacturer’s protocol.

### SNP genotyping

The extracted DNA was dissolved in TE buffer (10 mM Tris, 1 mM EDTA; pH = 7.8), quantitated by measuring the absorbance at 260 nm, and then stored at −20°C for genotyping. The primer was designed by Generunner 6.2.07 Beta software and synthesized by Beijing Liuhe Huada Gene Company. The genotype was completed by the PCR-restriction fragment length polymorphism (PCR-RFLP). The region of the GDF5 (+104T/C; rs143383) encompassing SNP was amplified using the TaqMan® 5′ allelic discrimination technique for amplifying and detecting specific polymorphisms in purified genomic DNA samples. The sense sequence was (5-AGCACACAGGCAGCATTACG-3), while the antisense sequence was (5-CCAGTCCCATAGTGGAAATG-3). The TaqMan® MGB probes/extension primers were (VIC AACTCGTTCTTGAAAGGAGAAAGCC) to detect the allele 1 sequence and (6FAM ACCGCCCCCTTTCTCCTGCACAACT) to detect the allele 2 sequence. The reaction system included 12 μl of 2× Es Taq MasterMix, 2 μl forward and reverse primers with 10 μl.mol/l, 2 μl DNA templates, and 9 μl. The PCR program consisted of the following steps: initial denaturation step at 94°C for 2 min, followed by 30 cycles: denaturation at 98°C for 10 s, annealing at 61°C for 30 s, extension at 72°C for 30 s, and extension again at 72°C for 2 min, and stored at 4°C. The PCR product was incubated overnight at 37°C with restriction enzyme. Then the mixtures were electrophoresed and visualized. The expected fragment was CC in 196 bp, 196,158, and 38 bp in AG, and 158 and 38 bp in TT.

### Statistical analysis

We used the SPSS 23.0 platform to complete all analysis. The quantitative data were expressed by using means ± S.D., and independent *t* test was used for comparison between case group and control group. The qualitative variables were expressed using count and percent, and Chi-square test was used for comparison between two groups. The Hardy–Weinberg equilibrium (HWE) was tested to evaluate whether the control group could represent the whole population by a goodness-of-fit χ^2^ test. The rude and adjusted ORs with 95% confidence intervals (CIs) were calculated to assess the relationship between *GDF5* gene and KOA. We used the stratification analysis to explore the interaction between gene and other factors including: age (<60 vs ≥60), gender (female vs male), history of smoking and drinking (Yes vs No), BMI (normal vs overweight or obesity), ESR (≥20 vs <20) and CRP (ESR ≥ 8 vs <8), and Kellgren–Lawrence grading (I/II vs III vs IV). *P*<0.05 was considered as a significant level.

## Results

Table 1 presented the clinical data of case group and control group. For case group, 44.8% of KOA patients were male and the ratio was 55.2% for females. The ratios of males and females were 46.6 and 53.4% in the control group, respectively. There was no significant difference in gender ratio between two groups (*P*=0.639). The mean age of case group and control group were 56.1 and 55.5 years, respectively. The rates of history of smoking and drinking were 3.29 and 28.9% in the case group, and 39.3 and 32.5% in the control group. No significant differences were observed (*P*=0.301, *P*=0.186). There was also no significance in course of disease between two groups (1.2 ± 0.8 vs 1.2 ± 0.9, *P*=0.102), and neither was BMI (*P*=0.595). The positive rates of ESR > 20 and CRP > 8 were 53.1 and 64.2%, respectively.

**Table 1:** Comparison of general characteristic between case and control groups

Factors	Case group (*n*=288)	Control group (*n*=397)	χ^2^/t	*P*
Gender			0.220	0.639
Male	129 (44.8%)	185 (46.6%)		
Female	159 (55.2%)	212 (53.4%)		
Age	56.1 ± 6.5	55.5 ± 6.3	−0.935	0.350
BMI	23.8 ± 3.2	23.9 ± 3.0	0.532	0.595
Course of disease	1.2 ± 0.8	1.2 ± 0.9	1.639	0.102
Smoking			1.069	0.301
Yes	102 (35.4%)	156 (39.3%)		
No	186 (64.6%)	241 (60.7%)		
Drinking			1.751	0.186
Yes	80 (27.8%)	129 (32.5%)		
No	208 (72.21%)	268 (67.5%)		
				
ESR ≥ 20	153 (53.1%)	-		
CRP > 8 mg/l	185 (64.2%)	-		
Kellgren–Lawrence grading		-		
I/II	155 (53.8%)			
III/IV	133 (46.2%)			
rs143383			28.110	0.000
TT	124 (43.1%)	206 (51.9%)		
CT	105 (36.5%)	159 (40.1%)		
CC	59 (20.5%)	32 (8.1%)		

### Association of GDF5/rs143383 gene polymorphism with KOA

Six hundred and eight-five blood samples were genotyped and the concordance rate of genotype was 100%. The genotype frequency was 43.1% for TT, 36.5% for CT, and 20.5% for CC in the case group; and 51.9% for TT, 40.1% for CT, and 8.1% for CC. The HWE test indicated no significant differences in the case group and in the control group (*P*>0.05). There was significant difference in genotype distribution between case group and control group (χ^2^ = 22.661, *P*=0.000). The minor C allele was significantly higher in the case group than that in the control group (20.5 vs 8.1%, *P*=0.000, OR = 1.62, 95% CI: 1.29–2.03). Significant differences were also observed in other gene models. The TT genotype and the TT/CT combination were more common in the case group than that in the control group. Compared with TT genotype, CC (OR = 3.06, 95% CI: 1.89–4.97) and CT/CC (OR = 2.94, 95% CI: 1.85–4.66) increased the risk of OA. The codominant model also shows the similar result (OR = 1.46, 95% CI: 1.07–1.98, *P*=0.016). We also conducted the multiple logistics to evaluate the relationship between rs143383 gene polymorphism and KOA risk by adjusting some potential factors including age, gender, smoking, drinking, BMI. The results were still significant in each model except the (TT vs CT: *P*=0.585 and 0.539). The results were shown in Table 2.

**Table 2:** Logistic regression analysis of associations between GDF5/rs143383 gene polymorphism and KOA

Genotype	Cases		Control		OR (95% CI)	*P*	aOR (95%CI)	*aP*
	*n*	%	*n*	%				
TT vs CC	124/59	43.1/20.5	206/32	51.9/8.1	3.06 (1.89–4.97)	0.000	3.25 (1.99–5.30)	0.000
TT vs CT	124/105	43.1/36.5	206/159	51.9/40.1	1.10 (0.79–1.53)	0.585	1.11 (0.80–1.55)	0.539
TT/CT vs CC	229/59	79.6/20.5	365/32	91.9/8.1	1.46 (1.07–1.98)	0.016	1.46 (1.07–1.98)	0.016
TT vs CT/CC	124/164	43.1/57.0	32/191	51.9/48.1	2.94 (1.85–4.66)	0.000	3.10 (1.95–4.94)	0.000
T vs C	353/223	61.3/38.7	571/223	71.9/28.1	1.62 (1.29–2.03)	0.000	-	-

Abbreviation: aOR: adjust OR, adjust age, gender, smoking, drinking, BMI.

### Stratified analyses

Subgroup analyses were performed in different ages (<60 vs ≥60), gender (female vs male), history of smoking and drinking (Yes vs No), BMI (normal vs overweight or obesity), ESR (≥20 vs <20) and CRP (ESR ≥ 8 vs <8), and Kellgren–Lawrence grading (I/II vs III vs IV). We first conducted subgroup analyses in different age, gender, weight, smoking, drinking. As shown in Table 3, significant differences were observed in TT vs CC, TT vs CT/CC, and A vs G for male and female (*P*<0.05), and others show no significant differences (*P*>0.05). For age, all models show significant differences (*P*<0.05) for those whose age was more than 60 years, and no significant difference was observed for those under 60 years. For non-smoking group, there were significantly differences between case group and control group, and for smoker, significant level was found in TT vs CC and allele gene models. Patients with drinking and BMI ≥ 24 also shows significant relationship between rs143383 and OA under the following models: TT vs CC (*P*=0.000, *P*=0.018), TT/CT vs CC (*P*=0.043), TT vs CT/CC (*P*=0.000, *P*=0.009), T vs C (*P*=0.024, *P*=0.000). Others gene model indicated no significance (*P*>0.05). We also explored the relationship between gene genotype and some inflammatory factors in the case group. The results indicated that inflammatory biomarkers and grading score were not associated with GDF gene polymorphism (*P*>0.05, Table 4).

**Table 3:** Subgroup analyses between GDF5/rs143383 gene polymorphism and the risk of KOA

Variables	rs143383 (Case/control)			TT vs CC		CT vs CC		TT/CT vs CC		TT vs CT/CC		T vs C	
	TT	CT	CC	OR (95% CI)	*P*	OR (95% CI)	*P*	OR (95% CI)	*P*	OR (95% CI)	*P*	OR (95% CI)	*P*
Sex													
Male	56/97	47/74	26/14	3.22 (1.55–6.66)	0.002	1.10 (0.67–1.80)	0.704	1.43 (0.91–2.26)	0.116	3.08 (1.54–6.17)	0.001	1.64 (1.17–2.30)	0.004
Female	68/109	58/85	33/18	2.94 (1.54–5.63)	0.001	1.09 (0.70–1.72)	0.697	1.42 (0.94–2.14)	0.099	2.82 (1.52–5.23)	0.001	1.76 (1.31–2.36)	0.000
Age (years)													
≥60	68/127	64/99	41/20	3.83 (2.08–7.05)	0.000	1.21 (0.79–1.86)	0.391	1.65 (1.11–2.45)	0.013	3.51 (1.97–6.24)	0.000	1.85 (1.39–2.48)	0.000
<60	56/79	41/60	18/12	2.12 (0.94–4.74)	0.069	0.96 (0.57–1.63)	0.964	1.16 (0.71–1.88)	0.558	2.15 (0.99–4.67)	0.053	1.31 (0.90–1.89)	0.159
Smoking													
Yes	36/86	41/52	25/18	3.31 (1.68–6.54)	0.000	0.82 (0.54–1.24)	0.335	1.10 (0.75–1.62)	0.611	3.63 (1.88–6.99)	0.000	2.05 (1.42–2.97)	0.000
No	88/120	64/107	34/14	3.32 (1.62–6.82)	0.001	1.88 (1.07–3.31)	0.028	2.25 (1.35–3.77)	0.002	2.49 (1.28–4.85)	0.007	1.41 (1.06–1.89)	0.019
Drinking													
Yes	34/65	32/48	14/16	4.41 (2.35–8.26)	0.000	1.03 (0.69–1.53)	0.883	1.46 (1.01–2.10)	0.043	4.35 (2.38–7.95)	0.000	1.54 (1.06–2.25)	0.024
No	90/141	73/111	45/16	1.67 (0.73–3.83)	0.224	1.28 (0.69–2.35)	0.436	1.37 (0.78–2.41)	0.268	1.50 (0.69–3.26)	0.309	1.77 (1.35–2.33)	0.000
BMI													
≥24	55/80	45/71	26/16	2.36 (1.16–4.81)	0.018	0.92 (0.56–1.53)	0.753	1.18 (0.75–1.89)	0.470	2.45 (1.25–4.81)	0.009	1.40 (1.00–1.98)	0.053
<24	69/126	60/88	33/16	3.77 (1.94–7.33)	0.000	1.25 (0.80–1.93)	0.329	1.63 (1.09–2.45)	0.018	3.42 (1.81–6.46)	0.000	1.80 (1.33–2.45)	0.000

**Table 4:** Comparison of genotypic and allelic frequencies of GDF5/rs143383 polymorphism amongst KOA subgroups stratified

Cases	TT	CT	CC	T	C
ESR ≥ 20	74 (48.4)	49 (32.0)	30 (19.6)	197 (64.4)	109 (35.6)
ESR < 20	50 (37.0)	56 (41.5)	29 (21.5)	156 (57.8)	114 (42.2)
*P*	0.261	0.050		0.105	
OR (95% CI)	0.70 (0.38–1.30)	0.59 (0.35–1.20)	1.00	0.76 (0.54–1.06)	1.00
CRP > 8 mg/l	83 (44.9)	68 (36.8)	34 (18.4)	234 (63.2)	136 (36.7)
CRP ≤ 8 mg/l	41 (39.8)	37 (35.9)	25 (24.3)	119 (57.8)	87 (42.2)
*P*	0.672	0.908		0.196	
OR (95% CI)	0.67 (0.35–1.27)	0.91 (0.53–1.57)	1.00	0.79 (0.56–1.13)	1.00
Grade I/II	64 (41.3)	61 (39.4)	30 (19.4)	189 (38.3)	121 (61.7)
Grade III/IV	60 (45.1)	44 (33.1)	29 (21.8)	164 (43.1)	102 (56.9)
*P*	0.327	0.923		0.866	
OR (95%CI)	0.77 (0.46–1.30)	1.03 (0.56–1.92)	1.00	1.03 (0.74–1.44)	1.00

## Discussion

The present study found that GDF5 gene polymorphism was associated with KOA. The TT allele gene can increase the risk of KOA. Our study first found that there were some interactions between GDF5 and age, smoking, drinking, and BMI for the increased risk of KOA. Our study provided more evidences for association between *GDF5* gene and KOA.

It is well acknowledged that the occurrence of KOA is determined by gene and environment and their interaction(s). Epidemiological and genetic studies have also shown that OA is a polygenic disease. Previous studies had screened several candidate genes associated with KOA in different populations [21]. However, the reports of most studies did not come to an agreement about identified susceptibility gene of KOA in different populations. GDF5 (+104T/C), located in the 5′-UTR of the *GDF5* gene, was one of the genetic variants that has garnered extensive attention as a genetic variant associated with OA. GDF5 (also known as cartilage-derived morphogenic protein 1, CDMP1) is one of the members of the TGF-β superfamily, which participates in the process development, repair, and maintenance of cartilage, bone, and soft tissues of the synovial joint [22]. The *GDF5* gene was found to be expressed in the articular cartilage of human adults and is involved in the processes of development, homeostasis, and repair of cartilage, bone, and articular cartilage [23]. One of the primary pathology symptoms of OA is the degeneration of articular cartilage, characterized by degradation and synthesis imbalance of chondrocytes, extracellular matrix, and subchondral bone that was usually caused by mechanical and biological factors. The articular cartilage of the knee is fibrous cartilage, and collagen in the cartilage matrix accounts for 50–60% (mainly type II collagen) [24]. The type II collagen is essential to maintain the mechanical strength of cartilage because the collagen can hold on the stress of cartilage. The destruction of collagen fibers in superficial articular cartilage is an important histopathological signal of early-stage OA. The experiments *in vivo* have suggested that the diameter of collagen fibers in early-stage OA articular cartilage increases, and the changes in the microstructure of usually occur earlier than subchondral bone destruction [25].

Miyamoto et al. [23] reported that the gene encoding GDF5 is associated with OA in Asian populations. A SNP in the 5′-UTR of GDF5 (+104T/C; rs143383) showed significant association (*P*=1.8 × 10^−13^) with hip OA in two independent Japanese populations. This study indicated that GDF5 as a susceptibility gene for OA and suggest that decreased GDF5 expression is involved in the pathogenesis of OA [23]. Tsezou et al. [26] assessed whether this SNP was also associated with KOA in a Greek Caucasian population sample. The +104T/C SNP was genotyped in a total of 519 case–control cohort; 251 patients with idiopathic KOA and 268 controls were used. No significant differences were found in genotype or allele frequencies of the +104T/C SNP of GDF5 gene between cases and controls (*P*>0.05). Also, no significant differences in allelic and genotypic frequencies were found when the individuals were stratified by sex. This study implied that the +104T/C; rs143383 GDF5 core promoter polymorphism is not a risk factor for OA etiology in Greek Caucasians. Tuluce et al. [23] assessed whether this SNP was also associated with OA in the Eastern Turkey population. A total of 172 cases including 95 patients with idiopathic OA and 77 control cases were recruited into the study. In terms of genotype comparison there was not any correlation between patient and control group. Frequency of C allele was found to be higher in patient group than control group and statistical analysis showed a poor correlation in allele frequencies of the +104T/C SNP of GDF5 gene between cases and controls (*P*>0.05) [27]. Mervat et al. [28] conducted a case–control study in Egypt populations, and no statistically significant association was detected on comparing the frequency distribution of allele and genotype frequencies of the SNP in patients and healthy controls. But the results of this study revealed a possible genetic association between GDF5 (+104T/C) SNP and the severity of KOA, which might be of benefit for the detection of patients with a high risk for disease progression [29]. These studies above have some limitations such as small sample size, and mixed study population with different OA. The present study only consisted of KOA patients with larger sample size.

Moreover, the discrepancy between the results of the present and previous studies performed worldwide may be attributed to the following several reasons. On one hand, these studies were conducted in different study populations. Different populations have different genetic backgrounds. Furthermore, our result indicated that there is some interaction between GDF5 gene and some environmental factors. Population from different areas have different eating habits. On the other hand, some studies were under the regulation level. They reported that the GDF5 expression was influenced by DNA methylation in human cell lines. The methylation of GDF5 locus is related to augmentation in rs143383 allele imbalance. Sub1, SP1, and SPP3, and DEAF transactivating proteins bind two alleles of rs143383 and repress their transcription [30]. Therefore, methylation regulates GDF5 expression in cartilage and regulates the functional effect of rs143383 by altering the binding of SP1 and SPP3 and DEAF transcriptional repressors [31]. Besides, the methylation level was different in different joint tissues. The types of OA could be attributed to the difference between previous results and our data. Furthermore, differences in genotyping methods and differences in patient’s inclusion criteria might be other factors yielding inconsistent results in different studies. Several study limitations must be addressed to the present study. One the of limitations is the study design. Just like previous study, the present study was based on case–control design. This type of study design has some weakness in identifying the cause–effect relationship. The other one is that the adjusted confounding factors are restricted. Some patients may have received some treatment such as hormones, which may exert some effects on results.

To sum it up, our results revealed a possible genetic association between GDF5 and KOA, and the TT genotype of rs143383 increased the risk of KOA in Chinese Han population. The interaction between *GDF5* gene and environment factors (smoking, drinking, and obesity) further increased the risk of KOA. Study with larger sample size and longitudinal design is required, and the future study should explore the specific molecular mechanism.

## Abbreviations

BMI: body mass index
CI: confidence interval
CRP: C-reactive protein
ESR: erythrocyte sedimentation rate
GDF5: growth and differentiation factor 5
HWE: Hardy–Weinberg equilibrium
KOA: knee osteoarthritis
OA: osteoarthritis
OR: odds ratio
SNP: single nucleotide polymorphism
TGF-β: transforming growth factor-β
